# Positive effects of brief body exercises on mood: An interventional EMA study

**DOI:** 10.1016/j.nsa.2026.106999

**Published:** 2026-04-06

**Authors:** Lea Nitzpon, Johanna Rehder, Richard Hohmann, Carlotta Kunst, Rika Gross, Claudio R. Nigg, Markus Reichert, Marcus Müller

**Affiliations:** a Department of Health Science, Institute of Sport Science, University of Bern, Switzerland; b eHealth and Sports Analytics, Faculty of Sport Science, Ruhr University Bochum, Germany; c Department of Psychiatry and Psychotherapy, Central Institute of Mental Health, Medical Faculty Mannheim, Heidelberg University, Germany; d Department of Sport and Exercise Science, Faculty of Natural and Life Sciences, University of Salzburg, Austria; e Institute for Physical Education and Sport, Karlsruhe University of Education, Germany

**Keywords:** Ambulatory assessment, Ecological momentary assessment, Physical activity, Affective well-being, Mood

## Abstract

Short bouts of physical activity in daily life are associated with affective well-being and may promote mental health. However, the causality and differential effects of distinct physical activity types remain unknown. This study investigated how different brief exercises (cardio, fascia training, breathing, progressive muscle relaxation, yoga, body awareness) influence three mood dimensions: energetic arousal, valence and calmness. Using an interventional Ecological Momentary Assessment (EMA) design, we assessed momentary mood states before and after brief smartphone-based exercise intervention in daily life of 100 participants (females = 52, males = 48, mean age = 32.83). We found overall positive within-person effects of the intervention on energetic arousal (b = 1.69, *p* = .06), valence (b = 1.80, *p* < .001) and calmness (b = 9.29, *p* < .001). Furthermore, results showed that within-person pre-intervention negative mood served as a significant moderator, i.e., energetic arousal increased (b = .19, *p* = .04), while the effect on calmness (b = .12, *p* = .06) remained stable across mood states. For valence, the benefit of the intervention slightly attenuated when momentary negative mood was high (b = −.11, *p* = .04). In addition, specific exercise types produced differential effects: energetic arousal increased most following cardio and fascia training, while calmness increased across all exercise types. Improvements in valence were most pronounced in breathing, yoga, and fascia training. These findings suggest that individualized exercise selection may differentially influence mood responses and highlight the potential of targeted, context-sensitive interventions to optimize affective well-being in daily life. If replicated in patient samples, the findings promise high value for the prevention and intervention of mental health, especially for affective disorders.

## 1 Introduction

Physical activity (PA) influences a range of biological and psychological processes, providing protective effects for cardiovascular, metabolic, and mental health (Cleven et al., 2020), (Silverman and Deuster, 2014), (Yu et al., 2025). One particularly important outcome is affective well-being, which is closely related to mental health outcomes, notably affective disorders (BfArM). Associations between PA and affective well-being have widely been demonstrated in the literature (Kanning et al, 2013), (Timm et al., 2024), (Reed and Ones, 2006), (Reed and Buck, 2009). Affective well-being is an umbrella term for affective states that may be differentiated into constructs such as emotions, moods and core affect (Dukes et al., 2021). In this study we investigate the effects of PA on mood. A positive emotional reaction to PA can predict both current and future PA engagement and is therefore hypothesized to be crucial for initiating and maintaining an active lifestyle (Cameron et al., 2018), (Rhodes and Kates, 2015), (Singh et al., 2023).

Throughout the day, a person's mood fluctuates in response to numerous stimuli. Subtle, short-lived shifts of feelings are difficult to capture with traditional retrospective questionnaires (Trull and Ebner-Priemer, 2013). Ecological momentary assessment (EMA) is a data collection method that enables intensive longitudinal sampling and allows for within-person analyses of momentary mood states in daily life (Reichert et al., 2020). EMA has overcome recall biases and opened new ways to understand how PA and mood interact in daily life (Reichert et al., 2020), (Reichert et al., 2017). Hence, this approach can reveal how mood fluctuates within individuals over time and proposes insights into possible temporal and causal links between PA and affective well-being (Trull and Ebner-Priemer, 2013).

In recent years, multiple EMA studies have investigated the effect of PA on mood (Timm et al., 2024), (Hollands et al., 2020), (Kanning and Schlicht, 2010), (Liao et al., 2017), (Liao et al., 2015). A dominant approach to capture mood via EMA is in the three-dimensional model comprising valence, energetic arousal, and calmness (Wilhelm and Schoebi, 2007). These different dimensions are often influenced differently by different types of activity (Reichert et al., 2017), (Schimmack and Grob, 2000). While PA has consistently been positively associated with energetic arousal and valence across various populations and conditions (Timm et al., 2024), (Benedyk et al., 2025), (Bossmann et al., 2013), (Giurgiu et al., 2024), (Kanning, 2013), non-exercise activity, such as walking or gardening, has been associated with reduced calmness and increased energetic arousal (Reichert et al., 2017), (Sudeck et al., 2018).

However, EMA studies on PA and mood have thus far primarily applied purely observational study designs and focused on naturally occurring activity and examining lagged associations between activity levels and subsequent well-being (Timm et al., 2024), (Kanning and Schlicht, 2010). While observational EMA designs are perfectly suited to maximize ecological validity of findings, they do not allow insights into the causality of associations. Experimental manipulation is a robust method to intentionally vary the independent variable, i.e., the intervention, by using randomization in an everyday setting. By controlling for contextual and external factors, this procedure isolates the intervention effects on the outcome variable, thereby strengthening causal inference. So far, studies have investigated incidental activity in everyday life in general but did not focus on specific activities (Reichert et al., 2017), (Benedyk et al., 2025), (Kanning, 2013). In contrast, the present study employs an interventional EMA design in which brief, standardized physical activity micro-interventions are actively prompted in daily life, and mood states are assessed immediately before and after each intervention. This design is rooted in a just-in-time adaptive intervention (JITAI) framework to deliver an intervention at the right moment based on an individual's current state or context (Klasnja et al., 2015), (Heron and Smyth, 2010). Consequently, immediate within-person effects can be attributed to specific physical activity interventions. We hypothesize that the brief, body-mindful movement programs of the Karlsruhe Relaxation Training (Karlsruher Entspannungs-Training, *ket*) are positively associated with within-person changes in momentary mood. Furthermore, we investigate whether momentary negative mood can be experimentally influenced in daily life through the *ket* program. To explore which forms of PA have different within-person effects on mood states, we included exercises spanning six domains: cardio, yoga, breathing, fascia training, progressive muscle relaxation, and body awareness. This allows us to test whether brief activities that target different physiological and psychological mechanisms differ in their within-person impact on momentary mood. Ultimately, this contributes to evidence-based strategies that could support mental health promotion in daily life.

## 2 Methods

### 2.1 Participants

The participants were recruited at the University of Education Karlsruhe through a motivational video in various lectures. Within the video, the study objectives and exercise selection were presented. After the students gave their voluntary consent by email, they were asked to recruit three to five acquaintances to also participate in the study. 109 individuals met the inclusion criteria: age 18+ and absence of chronic, endocrinological, cardiovascular, immunological and psychiatric disorders. Six individuals were excluded due to a compliance rate below 30% (less than two assessments per day), and three were excluded because of missing data resulting in a final sample of 100 participants.

### 2.2 Procedure

The study was conducted over ten consecutive days with participants completing eight daily assessments using an electronic diary (e-diary) provided on a study smartphone (Nokia C12). Time for the first and last assessment of the day could be selected by the participants individually. Additionally, at baseline, participants selected three preferred exercises from a total of 12 possible micro-interventions (duration approx. three minutes). Participants received 8 daily questionnaires to assess their current mood, scheduled every 2 h. Based on the participants e-diary data, i.e., energetic arousal <60 (VAS 0-100), up to four prompts were triggered per day; three exercise interventions and one control condition. The prompts were randomized within 1 day. Participants could postpone prompts for a maximum of 90 min. Each prompt remained active for 600 s; if unaddressed within this window, the prompt expired without further action. To ensure data integrity, the intervention counter only advanced when a prompt was completed. Ignored or expired prompts were not recorded as completed interventions. The control condition aimed at the working memory (based on the Great Brain Experiment) (Brown et al., 2014). One fifth of the participants received an intervention with three randomly assigned exercises. The exercises originate from the *ket*, which has been established at the Karlsruhe Institute of Technology within the Institute of Sports and Sports Science since 2020. *ket* develops, implements, and evaluates practical and everyday-suitable programs for physical education, stress management, and relaxation (Fessler, 2018), (Fessler, 2020), (Fessler, 2021) (Fessler and Müller, 2020) (Fessler et al., 2023), (Fessler and Müller, 2025). The six exercise domains each consist of two specific exercises and can be categorized based on movement intensity as follows: The cardio and fascia exercises are the most movement-intensive, followed by yoga and body awareness exercises. The breathing and progressive muscle relaxation exercises are comparatively low in movement. A detailed description of the exercises can be found in the Supplementary Material. As a control task participants completed the “Am I impulsive?” module from the Great Brain Experiment. In this task fruits fall from on a central tree wither on the left or right side. Participants were instructed to tap the falling fruits as quickly as possible when a cue (a circle) appeared. Some fruits appeared rotten and participants were then required not to tap the screen. This task requires sustained attention and rapid motor inhibition and serves as an active control task (Brown et al., 2014).

The ethics proposal for this study called ELVIS (Energize & distress your Life via digital short bouts exercises) was approved based on a similar research proposal by the Charité Berlin (EA 1/270/22, 16.02.2023) and Universitätsmedizin Mannheim (2023-536, 28.03.2023).

### 2.3 Measures

Mood states were assessed using the German version of the *Multidimensional Mood Questionnaire* (Wilhelm and Schoebi, 2007). The instrument consists of six bipolar items representing three mood dimensions: energetic arousal (EA), valence (V), and calmness (C). The items were EA1: tired - awake; EA2: without energy - full of energy, V1: content - discontent; V2: unwell - well, and C1: agitated - calm; C2: relaxed - tense (Wilhelm and Schoebi, 2007). Items were rated on a visual analogue scale (VAS) from 0 to 100. The VAS is the most reliable and validated scale for EMA assessment of mood (Haslbeck et al., 2025). Momentary negative mood was measured momentarily prior to each intervention/control prompt by using seven items for positive and negative affect (Myin-Germeys et al., 2003). The items were administered in German, translation is provided: “At the moment I feel …” 10.“… cheerful”, 11. “… lonely”, 12. “… energetic”, 13. “… anxious”, 14. “… down”, 15. “… guilty”, 16. “… irritable” on a Likert scale from 1 = does not apply at all to 7 = fully applies. For the ambulatory assessment participants were equipped with an Android study smartphone with the *movisensXS* application installed.

### 2.4 Statistical analyses

Linear mixed-effects (LME) models were used to analyse the data in *R* (package lme4). Therefore, the difference scores of each mood dimension were calculated as the score post-intervention minus the score pre-intervention. Person-mean centring was used for the predictor variable intervention and for the moderator momentary negative mood to distinguish between within- and between subject effects. This approach ensures that changes at the state level within individuals can be assessed independently of differences at the trait level between participants (Yaremych et al., 2023). To ensure that the mood measurements were taken in close proximity to the intervention, a lag filter was applied, including only assessments completed within 30 min after the intervention. To account for temporal dependencies typical for EMA data, hour of the day (continuous) and prompt number (continuous, representing the sequence of assessments) were included as covariates. These variables control for circadian fluctuations and potential reactivity or habituation to the repeated measurements. We also included age and sex as covariates in all models. We used random intercept multi-level models but included no random slopes thus focusing on average within-person effects. We did not consider autocorrelation in the covariance structure, but we controlled for temporal covariates (e.g., hour of the day and number of prompts). Regarding the covariance structure, we assumed a single variance component for the random intercept and an identity structure for the residuals. In the primary analysis, we tested the effects of the six *ket-*micro-interventions on the three mood dimensions Equation (1), here using the example of Energetic Arousal, describes the two-level LME to analyse momentary mood changes. Repeated measures *j* are nested within participant *i*. $\beta_{0}$ represents the intercept of the model and $\beta_{1-k}$ the fixed effects of the predictors. The within-person component (intervention_w) captures momentary deviations from an individual's average intervention exposure, whereas the between-person component (intervention_b) reflects differences in overall intervention exposure across participants. $u_{0i}$ represent the random intercept for participant *i* at level 2 and $e_{ij}$ the residuals at level 1.

Equation 1 $${Energetic\;Arousal}_{ij}=\beta_{0}+\beta_{1}{\left(intervention\_w\right)}_{ij}+\beta_{2}{\left(intervention\_b\right)}_{ij}+\beta_{3}{\left(age\right)}_{i}+\beta_{4}{\left(sex\right)}_{i}+\beta_{5}{\left(hour\;of\;day\right)}_{i}+\beta_{6}{\left(prompt\;number\right)}_{i}+u_{0i}+e_{ij}$$

We also tested whether pre-intervention negative mood moderated the intervention effects by including an interaction term between intervention (exercise vs. control) and within-person centred negative mood. Momentary negative mood was decomposed into within-person (negaff_w) and between-person (negaff_b) components. (see Eq. (2)).

Equation 2 $${Energetic\;Arousal}_{ij}=\beta_{0}+\beta_{1}{\left(intervention\_w\times negaff\_w\right)}_{ij}+)+\beta_{2}{\left(intervention\_b\times negaff\_b\right)}_{ij}+\beta_{3}\left({age}_{ij}\right)+\beta_{4}\left({sex}_{ij}\right)+\beta_{5}\left({hour\;of\;day}_{ij}\right)+\beta_{6}\left({prompt\;number}_{ij}\right)+u_{0i}+e_{ij}$$

A secondary, exploratory aim was to examine whether the effects of everyday physical activity on mood vary across the 12 individual *ket* exercises, representing different domains, i.e., cardio, yoga, breathing, fascia training, progressive muscle relaxation, and body awareness (see Eq. (3)). β_1_ denotes the fixed effects of exercise type at level 1, estimating differences in energetic arousal between exercise categories relative to the reference group. β_2_–β_7_ represent between-person effects indicating whether having a specific exercise included in one's assigned set was associated with mood

Equation 3 $${Energetic\;Arousal}_{ij}=\beta_{0}+\beta_{1}{\left(exercise\right)}_{ij}+{\beta_{2}{\left(Yoga\right)}_{i}+\beta_{3}{\left(Cardio\right)}_{i}+\beta_{4}{\left(Fascia\;Training\right)}_{i}+\beta_{5}{\left(Breathing\right)}_{i}+\beta_{6}{\left(PMR\right)}_{i}+\beta_{7}{\left(Body\;Awareness\right)}_{i}+\beta }_{8}{\left(age\right)}_{i}+\beta_{9}{\left(sex\right)}_{i}+\beta_{10}{\left(hour\;of\;day\right)}_{i}+\beta_{11}{\left(prompt\;number\right)}_{i}+u_{0i}+e_{ij}$$

## 3 Results

### 3.1 Descriptive statistics

Descriptive statistics for the final sample (N = 100, females = 52, males = 48) are presented in Table 1. The inter-item reliability (Cronbach's α) for the momentary mood dimensions was calculated across all measurement occasions. Reliability was good to excellent for all dimensions: energetic arousal, α: .80 (95% CI [.75, .77]); valence, α: .91 (95% CI [.93, .94]); and calmness, α: .87 (95% CI [.86, .87]). The intraclass correlation coefficient for negative mood was .56, indicating that a portion of the variance in negative mood was due to differences between participants rather than momentary fluctuations within individuals.

**Table 1:** Descriptive statistics.

Variable	Mean (SE)
Age [yrs.]	32.83 (16.86)
Mean compliance rate	.7 (.20)
Response frequency [s]	51.0 (14.20)
Average time spent on prompt [min]	14.68 (2.28)
Time lag pre-post assessment [min]	1.02 (.66)

*Note.* SE = standard error.

### 3.2 Effect of the intervention on the mood dimensions

**Fig. 1:**
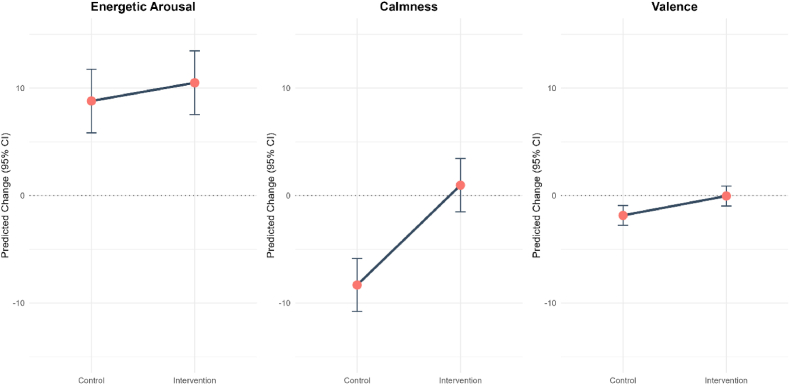
Within-person effect of the intervention on the three mood dimensions. The change score (pre-to post-intervention) is displayed for the control and the intervention condition.

A linear mixed-effects model was conducted to test the effect of the micro-intervention (vs. control) on the three mood dimensions, controlling for age, sex, hour of day, and prompt number. The micro-intervention showed a significant positive within-person effect on calmness and valence. Specifically, calmness increased by 9.29 points during intervention compared to the participants own control condition (b = 9.29, SE = .94, *p* < .001, 95% CI [7.45, 11.13]). Valence significantly increased by 1.80 points (b = 1.80, SE = .54, *p* < .001, 95% CI [.75, 2.86]). For energetic arousal the effect did not reach significance but showed a positive trend with an increase of 1.69 points (b = 1.69, SE = .89, *p* = .059, 95% CI [-.06, 3.45]), (see Fig. 1). At the between-person level, no significant differences in mood changes were found across all dimensions (energetic arousal: b = 14.60, SE = 11.60, *p* = .208; valence: b = 6.48, SE = 4.46, *p* = .147; calmness: b = 8.81, SE = 10.40, *p* = .400).

### 3.3 Interaction effect between intervention and momentary negative mood

**Fig. 2:**
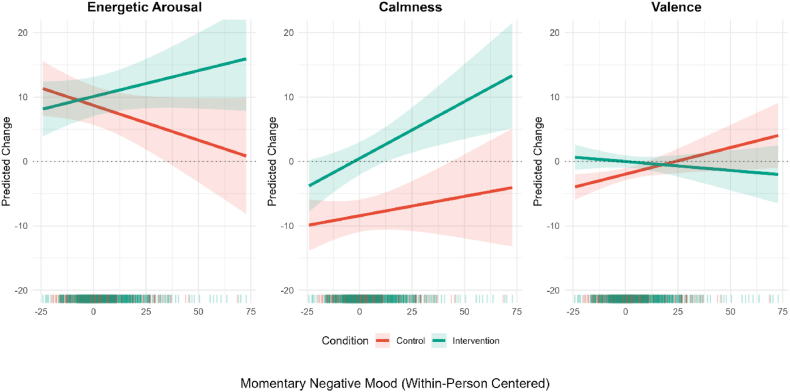
Predicted within-person change scores (post-pre intervention) for energetic arousal, calmness and valence as a function of pre-intervention negative mood. A value of 0 represents the participant's typical mood state. Shaded areas represent 95 % CI. Distribution of the data is displayed at the bottom.

The interaction between intervention and momentary negative mood was tested for each mood dimension using an LME (see Fig. 2). The intervention showed a significant stronger within-person effect on energetic arousal if momentary negative mood prior to the intervention had been higher (b = .19, SE = .09, *p* = .04, 95% CI [.01, .36]). A similar trend was observed for calmness, though it did not reach statistical significance (b = .12, SE = .09, *p* = .21, 95% CI [-.06, .30]). In contrary, the intervention within-person effect on valence was significantly negative (b = −.11, SE = .05, *p* = .04, 95% CI [-.21, −.01] .04). At the between-person level, no significant interaction was found (energetic arousal: b = −.14, SE = 1.49, *p* = .926; calmness: b = 2.48, SE = 1.30, *p* = .059; valence: b = .45, SE = .51, *p* = .377).

### 3.4 Exploratory analysis to investigate the effect of the different exercises on mood

**Fig. 3:**
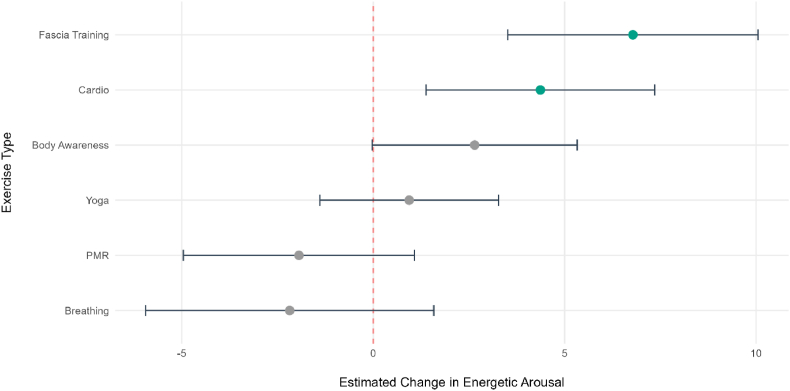
Comparative Effectiveness of the different *ket* exercises on within-person energetic arousal. The forest plot displays the fixed-effect estimates (b) for each exercise type compared to the control condition (red line). Error bars represent 95% CI. Green points indicate significant differences from the control condition (*p* < .05).

**Fig. 4:**
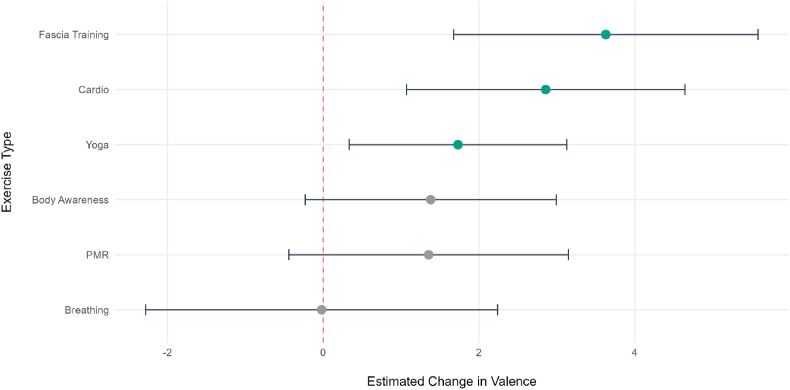
Comparative Effectiveness of the different *ket* exercises on within-person valence. The forest plot displays the fixed-effect estimates (b) for each exercise type compared to the control condition (red line). Error bars represent 95% CI. Green points indicate significant differences from the control condition (*p* < .05).

**Fig. 5:**
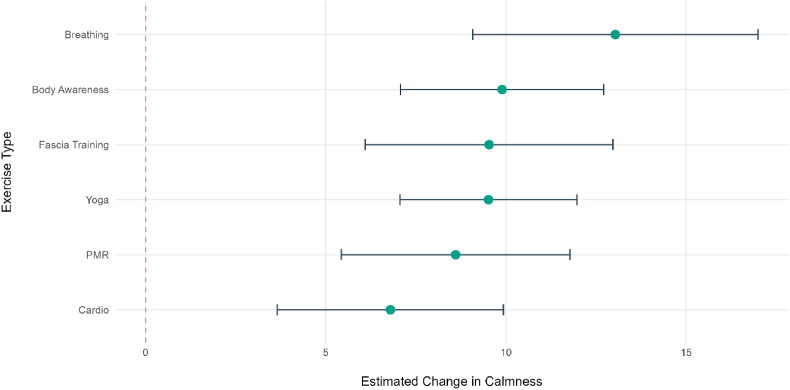
Comparative Effectiveness of the different *ket* exercises on within-person calmness. The forest plot displays the fixed-effect estimates (b) for each exercise type compared to the control condition (red line). Error bars represent 95% CI. Green points indicate significant differences from the control condition (*p* < .05).

An exploratory linear mixed-effects model was conducted to compare the within-person change in energetic arousal for each micro-intervention against the control condition (n = 100, 1376 data points), see Fig. 3, Fig. 4, Fig. 5 (detailed description of the pre- and post means of the mood dimensions stratified by exercise type in the Supplementary Material).The control condition was used as a reference and showed a significant increase in energetic arousal (b = 23.25, SE = 4.63, *p* < .001, 95% CI [14.37, 8.38]).When comparing the exercises against the control condition, fascia training showed the largest significant increase in energetic arousal (b = 6.78, SE = 1.67, *p* < .001, 95 % CI [3.51, 10.03]). Similarly, cardio (b = 4.37, SE = 1.52, *p* = .004, 95 % CI [1.39, 7.35]) also resulted in significantly greater increases in energetic arousal. In contrast, the effects of body awareness (b = 2.65, SE = 1.37, *p* = .053, 95 % CI [-.03, 5.32]), breathing (b = −2.18, SE = 1.92, *p* = .26, 95 % CI [−5.92, 1.59]), PMR (b = −2.94, SE = 1.54, *p* = .21, 95 % CI [−4.96, 1.06]), and yoga (b = .94, SE = 1.19, *p* = .43, 95 % CI [−1.37, 3.30]) were not significantly different from the control condition, suggesting they did not contribute to a greater increase in energetic arousal.

For valence, the LME model also used the control condition as the reference (n = 100, 1378 data points). The model indicated a significant baseline decline in valence for the control (b = −3.26, SE = 1.55, *p* = .04, 95 % CI [−6.11, −.31]). Fascia (b = 3.63, SE = 1.00, *p* < .001, 95 % CI [1.68, 5.57]), cardio (b = 2.86, SE = .91, *p* = .002, 95 % CI [1.09, 4.65]), and yoga (b = 1.73, SE = .71, *p* = .02, 95 % CI [.33, 3.11]) resulted in a significantly greater increase in valence compared to the control. Furthermore, body awareness (b = 1.38, SE = .82, *p* = .09, 95 % CI [-.23, 2.99]), PMR (b = 1.35, SE = .92, *p* = .14, 95 % CI [-.40, 3.17]) and breathing (b = −.02, SE = 1.15, *p* = .99, 95 % CI [−2.27, 2.24]) showed effects that were not significant.

For calmness, the model showed the strongest baseline effect (n = 100, 1377 data points): the control condition was associated with a highly significant decrease in calmness (b = −2.12, SE = 4.21, p = .61, 95 % CI [−10.37, −5.47]) during the assessment interval. All six micro-interventions significantly mitigated or reversed this decline when compared to the control reference. The largest positive effects were observed for breathing (b = 13.04, SE = 2.02, *p* < .001, 95 % CI [9.09, 17.00]), body awareness (b = 9.89, SE = 1.44, *p* < .001, 95 % CI [7.08, 12.71]), yoga (b = 9.52, SE = 1.25, *p* < .001, 95 % CI [7.07, 11.97]), and fascia (b = 9.53, SE = 1.76, *p* < .001, 95 % CI [6.12, 12.99]). Even the exercises with the smallest comparative effect, PMR (b = 8.61, SE = 1.62, *p* < .001, 95% CI [5.44, 11.77]) and cardio (b = 6.79, SE = 1.60, *p* < .001, 95 % CI [3.71, 9.98]), still resulted in a highly significant improvement in calmness relative to the control condition.

We also investigated sex- and age-specific differences influencing the selection of the specific exercises (see Table 2). Participants who selected fascia training had a higher mean age and unequal sex distribution was most pronounced for yoga and fascia training. Overall, yoga was the most frequently selected exercise and breathing the least.

**Table 2:** Descriptives for specific exercises.

Intervention	N	Prompts M(SD)	Max Prompts	Participants in Group	Age M	Males [%]	Females [%]
Control	365	3.72 (2.21)	9	98	33.1	41.5	58.5
Body Awareness	188	4.09 (2.28)	8	46	30.4	43.1	56.9
Breathing	90	3.61 (2.14)	7	25	33.0	48.2	51.8
Cardio	153	4.51 (3.21)	15	34	34.2	50.3	49.7
Fascia Training	124	4.60 (3.10)	15	27	37.8	26.6	73.4
PMR	163	5.09 (3.50)	14	32	32.4	50.9	49.1
Yoga	361	5.39 (4.18)	28	67	32.0	35.9	64.1

*Note.* M = mean, SD = standard deviation.

To examine whether individual characteristics moderated the effectiveness of the micro-interventions, interaction analyses with sex and age were conducted for all mood dimensions. For energetic arousal, the result showed a significant interaction between intervention and sex (b = 4.21, SE = 1.85, *p* = .02). Interaction between age and the intervention did not yield significant effects. For calmness, no significant interactions were found for sex nor for age. This indicates a universal calming effect across the study population. For valence, a significant interaction between the intervention and age was observed (b = −.08, SE = .03, *p* = .02).

### 3.5 Interaction effect between specific exercises and negative mood

We also tested whether pre-intervention negative mood did influence the respective exercises for any mood dimension. We did not find any significant effects.

## 4 Discussion

This study demonstrated positive within-person effects of brief physical activity exercises from the *ket* program ((Fessler, 2018), (Fessler, 2020), (Fessler, 2021) (Fessler and Müller, 2020) (Fessler et al., 2023), (Fessler and Müller, 2025)) across the three mood dimensions: energetic arousal, valence and calmness. Momentary negative mood moderated the interventions effects, such that higher negative mood predicted stronger improvements in energetic arousal. The intervention effect on calmness remained stable, regardless of the participant's mood. For valence, the intervention was less effective when negative mood was high. Finally, the different types of exercises yielded distinct effects on mood response: cardio increased energetic arousal most, whereas breathing showed the largest increase in calmness. These findings align with previous research demonstrating that physical activity is positively associated with affective well-being (Kanning, 2013), (Basso and Suzuki). Energetic arousal is consistently reported to increase after PA (Timm et al., 2024), (Benedyk et al., 2025), (Giurgiu et al., 2024). This immediate positive response likely reflects physiological activation processes that occur during PA (Bossmann et al., 2013). For valence, the observed increase also coincides with current literature, specifically for low to moderate PA (Benedyk et al., 2025), (Kanning, 2013). While some studies report reductions in calmness following exercise which is often attributed to a lack of autonomy regarding the timing or type of activity (Reichert et al., 2017) our results showed protective effects on calmness. This may be due to the *ket* program design where participants could choose the exercises leading to a more self-directed setting.

Beyond these general effects, a secondary analysis revealed that negative mood prior to the intervention moderated the intervention effect. Higher negative mood increased subsequent positive mood changes more strongly in energetic arousal. A significant negative moderation effect was observed for valence indicating that with greater negative mood the intervention was less effective. In particular, the intervention effect diminished as negative affect increased above the person-mean (i.e. +10 points above the person-mean) This suggests that the intervention may not be effective when negative mood is above a person's mean. However, only 23.75% observations exceeded negative affect > +10, therefore these results should be interpreted cautiously. Future JITAIs could profit from threshold-based triggering, i.e., only trigger when negative mood is below a certain threshold. Importantly, dimension-specific decision rules (i.e., for energetic arousal, calmness, valence) could be applied since the data showed differences across the dimensions. Previous findings indicate that the beneficial effects of PA on valence are strongest when the behaviour is fully autonomous (Rose and Parfitt, 2012). In our study the type of exercise was self-selected, but the timing was externally imposed.

We found that the different *ket* exercises yielded varying effects across the six exercise domains: yoga, breathing, PMR, fascia training, cardio and body awareness. When comparing the interventions against the control condition, only higher-intensity exercises such as cardio and fascia training led to significantly higher energetic arousal. Calmness increased across all exercises. The strongest effect was observed in breathing and the smallest in cardio which indicates that low-intensity, relaxation-focused activities are most effective for enhancing calmness (Ghandeharioun and Picard, 2017), (Narasimhan et al., 2011). In valence, the exercises also revealed positive effects compared to the control, but the extent was least across the three mood dimensions. Notably, both calmness and valence showed a significant decrease in the control condition whereas energetic arousal increased. This increase in energetic arousal within the control group may be attributed to the physiological and cognitive demands of the reactive control task. In contrast, the decrease in calmness and valence may be explained by the specific nature of the reactive control condition which was likely perceived as cognitively demanding. Such tasks require high levels of concentration, which can lead to an increase in physiological stress and therefore a reduction in calmness and valence (Tran et al., 2021).The variations across the mood dimensions may be explained by distinct physiological and psychological mechanisms (Basso and Suzuki). The immediate increase in energetic arousal following exercise may be primarily driven by the sympathetic nervous system (SNS). SNS activation during acute exercise triggers the release of neurotransmitters such as dopamine and norepinephrine which elevate heart rate and blood flow (Chu et al., 2025). Benedyk et al. (2025) showed that particularly higher levels of PA are associated with higher levels of energetic arousal (Benedyk et al., 2025). In contrast, calmness is associated with the activation of the parasympathetic nervous system (PNS) (Chu et al., 2025). Low-intensity and relaxation exercises like breathing and yoga, increase PNS activity by enhancing vagal tone, which regulates bodily functions like heart rate (Gerritsen and Band, 2018). Valence is a mood dimension influenced by complex psychological processes, such as sense of ability or accomplishment (Rose and Parfitt, 2010). Dissociative thoughts, i.e. concentrating on external stimuli as opposed to internal feelings, and being outdoors are factors that enhance valence response to PA (Bourke et al., 2021). In contrast, exercises within the *ket* program, i.e., PMR, body awareness, yoga and breathing, focus primarily on bodily sensation rather than external stimuli. Beyond these general considerations, Bourke et al. (2021) argue that inter-individual differences may stem from an individual's intrinsic motivation toward PA (Bourke et al., 2021). Individuals with greater intrinsic motivation will potentially have a more enjoyable PA experience. Finally, sex moderated the response to the intervention regarding energetic arousal which may indicate distinct physiological or psychological processing of physical interventions between men and women (Tamres et al., 2002), (Kudielka and Kirschbaum, 2005). Age showed a moderating effect on valence which could suggest that a shift in mood is easier obtained with younger age as changes of somatic perception with increasing age may dampen the positive effect (Pfeifer and Cawkwell, 2025).

The effectiveness of the specific exercises was not moderated by momentary negative mood. This suggests that the exercises were robust and not influenced by participant's mood states.

### 4.1 Limitations

This study has some limitations that warrant discussion. This study did not assess prior PA experience or current activity level. Therefore, it is not possible to determine whether our observed effects are due to fitness level or familiarity with specific exercises. Furthermore, the possibility for participants to freely select their exercises introduces a potential for preference bias. Given that sex-specific preferences for certain types of activity were observed, it is probable that familiarity with certain exercise types facilitate stronger mood response (Ekkekakis et al., 2005), e.g. familiarity with yoga could lead to a stronger calmness response. While this freedom of choice of short exercise types may limit strict causal interpretation, as the effects cannot be entirely disentangled from participant motivation, it significantly increases the ecological validity of the findings. This study design reflects how individuals naturally engage with exercises in daily life and makes the results more applicable to real-world behaviour. Another limitation is the variable compliance rate across the study population. Although we controlled for differences in engagement, participants with lower engagement might differ systematically, e.g.in motivation or stress level from those with higher engagement. This may limit the generalizability. At the same time, differing engagement levels provide insight into the real-world feasibility of mobile interventions. A methodological limitation is the use of an active control condition, a working memory task. This task might induce cognitive load and influence the mood dimensions, specifically contribute to the decline in calmness and thereby inflate relative intervention effects Using a passive control (e.g., time passing) could isolate the benefits of the exercise from the effects of cognitive load experienced during the control condition. Nevertheless, an active control condition can strengthen internal validity and ensure that observed effects are not due to doing anything. Our study adds to recent evidence revealing that momentary interventions show positive proximal outcomes and short-term improvement in mental well-being (Von Lützow et al., 2025), and it further supports the argument that the effectiveness of JITAIs depends critically on delivery timing, as susceptibility to intervention triggers may vary across mood states. However, our study did not investigate long-term effects of JITAIs. Since findings on sustainability of effects remain mixed in the literature (Von Lützow et al., 2025), (Nahum-Shani and Murphy) there is a need for future longitudinal studies.

### 4.2 Conclusion and implications

In summary, we found positive effects of physical activity on overall mood and significant moderation by pre-intervention negative mood. This supports the practical feasibility of selecting and delivering micro-interventions at optimal moments to maximize their effectiveness. The concept of JITAIs uses an individual's context, such as mood or activity level to deliver targeted interventions. The interventional EMA design captures real-time changes in momentary mood states, thereby allowing differentiation between intra-individual and inter-individual effects. Finally, these results may have clinical relevance for individuals with affective disorders, such as Major Depressive Disorder, if replicated in patient samples. Overall, our study emphasizes that even short, focused exercises can serve as effective tools for self-regulation and maintenance of affective well-being.

## Declaration of generative AI and AI-assisted technologies in the writing process

During the preparation of this work the author(s) used GPT-4o (OpenAI) in order to refine code for data analysis and to improve fluency and readability of the text. After using this tool/service, the author(s) reviewed and edited the content as needed and take(s) full responsibility for the content of the published article.

## Funding

The project was supported by the German Research Foundation through the Collaborative Research Center TRR265, project C05 (to MR; project grant no 402170461) and by the ERA-NET NEURON Project MASE (BMBF, 01EW2404A, to MR and SNSF 32NE30_219090 to CN).

## Declaration of competing interest

The authors declare that they have no known competing financial interests or personal relationships that could have appeared to influence the work reported in this paper.
