# Supplementary material for: Positive effects of brief body exercises on mood: An interventional EMA study
Source: Neurosci Appl. 2026 Apr 6;5:106999. doi: 10.1016/j.nsa.2026.106999 (PMC13091315; doi:10.1016/j.nsa.2026.106999)
Supplement: Multimedia component 1 [file mmc1.docx]

**Supplementary Material**

**Breathing exercises**

**Breath expansion** (duration: up to 2 minutes)

- Close your eyes to concentrate. Pay attention to your breath as it flows in and out evenly.
- Now change your breath lengths during inhalation and exhalation: First inhale quickly, then exhale slowly and prolonged.
- Now inhale long and exhale short.
- Now inhale and exhale slowly.
- Deepen your breath by counting slowly to four while inhaling and also while exhaling.
- Inhale slowly to four again, but count to five while exhaling, so that your lungs empty even more.
- Finally, breathe normally in and out again.

**Inspiration and Expiration** (duration: up to 2 minutes)

- Inhaling, raise your arms upwards. Then, exhaling evenly and deeply, slowly bring your arms down. Continue the movement. Note that breathing becomes easier and the breath deepens as your upper body straightens and your chest expands.
- Now interlace your fingers behind your head and stretch your elbows wide to the sides. Additionally, draw your navel towards your spine to create abdominal tension.
- With the inhalation, now stretch your thoracic spine by slightly tilting your shoulder girdle back. Hold this position for several more breaths.
- Make sure to tilt your head back only as far as you can continue to breathe calmly. This position should be comfortable in any case.
- With the last exhalation, slowly return to an upright position. Lower your arms and shake them out loosely.

**Progressive Muscle Relaxation exercises**

**Strengthen your Back** (duration: up to 2 minutes)

- I sit upright on the front third of the chair and place my hands on my thighs.
- I focus my attention on my lower back and pelvis. I feel into both and breathe in and out smoothly.
- Now I inhale and tilt my pelvis forward. My back straightens, and the back muscles become taut. A slight swayback may occur.
- I hold the tension for several more breaths. With each inhale, the sternum rises a little higher. The head stays in line with the spine. I feel the tension on both sides of the spine. My pelvic floor is also tense.
- Now I let go of all tension with the next exhale. I feel the release in my back and pelvis.
- For several breaths, I concentrate on my entire back. I notice how the relaxation spreads with each incoming breath. I feel how relaxed the muscles of my back and pelvis are.

**Pull in your belly** (duration: up to 2 minutes)

- I sit upright on the front third of my chair and bring my attention to my abdomen. I feel how it rises and falls with every breath.
- Now, as I inhale, I push my abdomen outward and hold my breath. Then I tense my abdominal muscles so they become firm and hard. The abdomen may draw in slightly again during this process.
- I consciously perceive the tension in my abdominal wall for as long as I feel comfortable. Exhaling, I release the tension in the abdominal muscles. I continue to breathe smoothly.
- Now, with the next exhalation, I gently draw my abdomen in so that the navel moves towards the spine. I hold the tension and continue to breathe smoothly. I feel the tension in my abdominal muscles and hold this tension for one more breath.
- Now, with the next exhalation, I let go of all tension. I feel the releasing sensation in the abdominal muscles and the abdominal cavity.
- For several breaths, I feel deeply into my abdominal cavity. I feel how the relaxation spreads throughout my abdomen. With every further breath, I let go a little more and calm my mind.

**Body Awareness exercises**

**Straighten your Back** (duration: up to 2 minutes)

-
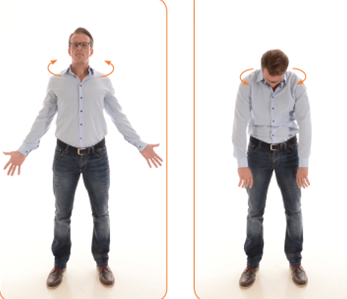

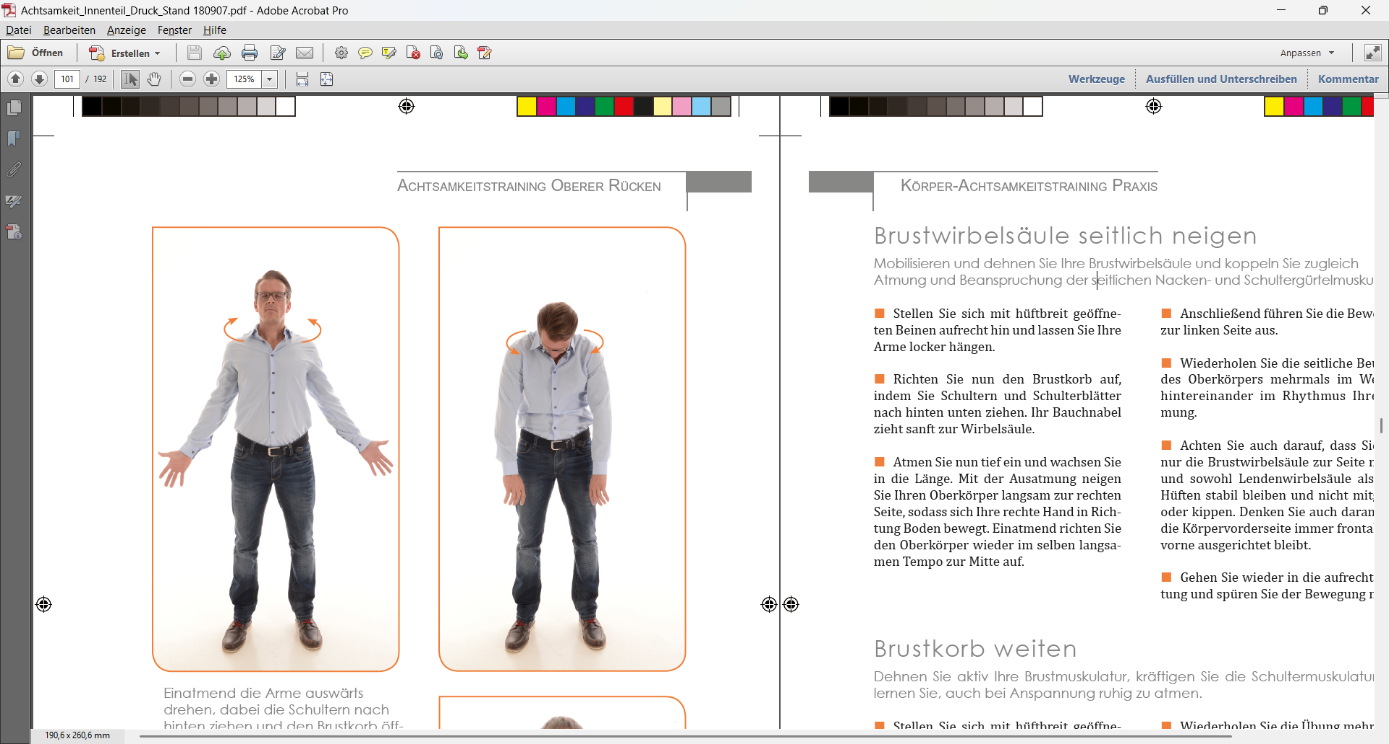
Take a stance with your feet hip-width apart and look straight ahead. Your arms hang loosely at your body.
- Inhale as you pull your shoulder blades together and rotate your arms so that your thumbs point outward.
- Exhale as you release the tension in your shoulders. Your upper body and head lean forward, turning your thumbs inward towards your body.
- Repeat the rising and lowering in a flowing motion several times.
- Inhale to straighten your head and upper body while opening your chest. Feel how liberating it is to straighten up, as posture and psyche are interconnected.

**Rotate the Chest** (duration: up to 2,5 minutes)

- Sit up straight in the front third of a chair and cross your right leg over your left. Now place your left hand on the outside of your right thigh and grab the seat of your chair with your right hand just behind your buttocks.
- Take a deep breath. As you exhale, slowly turn your upper body and head to the right. Stay in this position and continue to breathe calmly. With each exhalation, apply slight pressure on your thigh with your left hand by pulling gently, allowing yourself to rotate a little more.
- Release the tension in your left hand and gently turn your upper body and head back to the center.
- Place the bent leg on the ground and pause for two breaths. Then switch sides and perform the exercise by turning your upper body and head to the left now. Do not force anything: depending on your daily condition and limitations, you can challenge yourself more or less and adjust the intensity of the turn.
- Put the bent leg back down and savor the exercise.

**Yoga exercises**

**Business Dog** (duration: up to 2,5 minutes)

- Stand at the edge of a table and place your hands shoulder-width apart with your palms resting on the table surface.
- Step backward in small steps until your upper body is parallel to the ground. Your legs are initially bent, as the focus is on keeping the upper body extended.
- Hold this position for several breaths.
- Once you have settled into the position and feel comfortable, you can try to extend your legs a bit more with each exhalation. At the same time, you can try to extend your upper body a bit more with each exhalation. Your pelvis pushes back while the tailbone points toward the ceiling, lifting the lower back.
- Hold this position for several breaths as well. Breathe calmly and feel the intense stretch not only in your legs but also in your lower and upper back.
- Finally, return to the edge of the table with small steps and reflect on the exercise while standing upright.

**Upright Dancer** (duration: up to 2,5 minutes)

- Stand upright and focus your gaze on a point in front of you. Now shift your weight onto your left leg and stabilize yourself so that you can lift your right leg off the ground and gently bring your heel towards your buttock. Hold your ankle with your right hand. For stabilization, you can place your left hand on your hip or secure a stable stance by holding onto a chair back with your left hand.
- Stay in the position for several breaths and feel the stretch in your hip and thigh. Notice how the intensity of the stretch decreases with each breath.
- Now activate your abdominal muscles. Maintain the tension, as it protects against overstretching your lower back. Then lift your right foot backwards and upwards so that you enter a bow tension that stretches the entire front of your body.
- Stay here for several breaths and feel into your right hip joint, your spine, and your right shoulder.
- Now switch sides by performing the sequence with your right leg as the standing leg.
- Return to the upright position. Feel into the position for several breaths. Then relax both legs.

**Fascial exercises**

**Open Chest** (duration: up to 2,5 minutes)

- Stand upright with your feet shoulder-width apart. Let your arms hang relaxed by your sides.
- First, build tension along your arms by stretching your fingers. Then raise your outstretched arms in front of your body upwards and backwards into a 'V-shape' until you feel a stretch in your torso. Hold the position for a few breaths.
- Now intensify the stretch by gently leaning your upper body backwards. Keep your head in alignment with your spine. Consciously breathe into the stretch. Actively perceive how your sternum is directed forward and up, and how your ribcage expands a little more with each breath.
- Now continue the exercise by gently bouncing into the stretch with your outstretched arms.
- Return to an upright position, and finally savor the pleasant stretch.

**Swing the upper body forward and backward** (duration: up to 2,5 minutes)

- Stand upright and relaxed with your legs open wider than shoulder-width. Let your arms hang loosely by your sides.
- Inhale and raise your arms forward over your head until they are stretched. Now bring your body into a pre-tension by slightly tilting your upper body back.
- With the next exhale, bring your upper body forward towards the floor with your arms and swing your arms back through your legs. Inhale as you swing back into the stretch.
- Repeat the flowing movement as dynamically as possible. Bounce the movement loosely with your knees and use the catapult effect of the backward swing.
- Come back to an upright position. Stand relaxed and upright again, and feel the exercise for a few breaths.

**Cardio exercises**

**X-Swing** (duration: up to 2 minutes)

- Stand shoulder-width apart, your knees are slightly bent. Extend both arms forward to shoulder height. The palms face down.
- Inhaling, swing both arms dynamically sideways until slightly behind your body. At the same time, push off your toes.
- Exhaling, swing your arms back beyond the starting position, so that the arms cross in front of the center of your body and form a horizontal X. While doing this, rock back from your toes to the entire sole of your foot.
- Repeat the X-Swing several times.

**Reverse Fly** (duration: up to 2 minutes)

- Stand with your feet hip-width apart, knees slightly bent. Lean your upper body forward at about a 45° angle. Keep your back straight to reduce the load on the intervertebral discs. Bend your arms at a 90° angle in front of your body so that your palms face each other.
- Inhale, pull your shoulder blades together, and then lift your bent elbows above shoulder height.
- Exhaling, return to the starting position.
- Repeat the Reverse Fly several times in rhythm with your breath.

Table 1. Pre- and post means of the three mood dimensions stratified by exercise type

| Intervention | N_obs_ | Energetic Arousal M(SD) | Valence M(SD) | Calmness M(SD) |
| --- | --- | --- | --- | --- |
|  |  | Pre /  Post | Pre /  Post | Pre /   Post |
| Control | 363 | 41.71 (17.16) /  49.23 (20.65) | 50.44 (6.69) /  48.22 (8.97) | 72.23 (18.96) /  63.03 (22.59) |
| Body Awareness | 186 | 39.68 (15.43) /  49.70 (17.37) | 50.30 (5.66) /  49.57 (7.17) | 69.49 (20.02) /  70.16 (19.51) |
| Breathing | 88 | 38.91 (17.57) /  45.25 (19.46) | 52.43 (7.34) /  50.53 (8.78) | 71.23 (24.48) /  73.76 (22.50) |
| Cardio | 153 | 39.18 (15.74) /  49.82 (18.36) | 50.12 (8.08) /  50.82 (7.47) | 67.52 (20.63) /  67.05 (18.48) |
| Fascia Training | 123 | 40.24 (16.36) /  55.51 (19.14) | 49.02 (6.86) /  49.95 (5.44) | 68.18 (19.79) /  69.20 (18.68) |
| PMR | 159 | 40.92 (16.72) /  45.07 (18.50) | 50.44 (7.60) /  49.85 (6.33) | 72.25 (18.98) /  70.73 (18.77) |
| Yoga | 343 | 41.18 (17.88) /  51.14 (20.36) | 50.49 (6.79) /  49.85 (6.67) | 73.58 (21.81) /  74.51 (19.93) |

Table 2. Overall model results for Energetic Arousal

| term | estimate | SE | statistic | df | *p* | CI_low | CI.high |
| --- | --- | --- | --- | --- | --- | --- | --- |
| (Intercept) | 7.36 | 9.45 | 0.78 | 185.95 | 0.44 | -11.28 | 26 |
| intervention_centered | 1.69 | 0.89 | 1.89 | 1264.38 | 0.06 | -0.06 | 3.45 |
| intervention_mean | 14.63 | 11.58 | 1.26 | 198.71 | 0.21 | -8.21 | 37.47 |
| age | 0.05 | 0.06 | 0.86 | 75.93 | 0.39 | -0.07 | 0.17 |
| sex | -1.56 | 1.87 | -0.83 | 78.53 | 0.41 | -5.29 | 2.17 |
| hour_of_day | -0.35 | 0.09 | -3.98 | 1363.72 | 0 | -0.52 | -0.18 |
| prompt_number | -0.05 | 0.02 | -2.45 | 1330.75 | 0.01 | -0.09 | -0.01 |

Table 3. Overall model results for Valence

| term | estimate | SE | statistic | df | *p* | CI_low | CI.high |
| --- | --- | --- | --- | --- | --- | --- | --- |
| (Intercept) | -4.46 | 3.56 | -1.25 | 243.68 | 0.21 | -11.47 | 2.55 |
| intervention_centered | 1.8 | 0.54 | 3.35 | 1299.52 | 0 | 0.75 | 2.86 |
| intervention_mean | 6.48 | 4.46 | 1.45 | 263.8 | 0.15 | -2.3 | 15.26 |
| age | 0 | 0.02 | -0.04 | 67.57 | 0.97 | -0.03 | 0.03 |
| sex | 0.05 | 0.51 | 0.09 | 70.78 | 0.93 | -0.98 | 1.07 |
| hour_of_day | -0.14 | 0.05 | -2.84 | 1097.13 | 0 | -0.23 | -0.04 |
| prompt_number | 0.02 | 0.01 | 2 | 1360.8 | 0.05 | 0 | 0.05 |

Table 4. Overall model results for Calmness

| term | estimate | SE | statistic | df | *p* | CI_low | CI.high |
| --- | --- | --- | --- | --- | --- | --- | --- |
| (Intercept) | -5.56 | 8.47 | -0.66 | 167.79 | 0.51 | -22.29 | 11.16 |
| intervention_centered | 9.29 | 0.94 | 9.91 | 1247.8 | 0 | 7.45 | 11.13 |
| intervention_mean | 8.81 | 10.45 | 0.84 | 182.51 | 0.4 | -11.8 | 29.42 |
| age | 0.06 | 0.05 | 1.29 | 59.48 | 0.2 | -0.03 | 0.16 |
| sex | -2.27 | 1.53 | -1.48 | 61.95 | 0.14 | -5.33 | 0.79 |
| hour_of_day | -0.1 | 0.09 | -1.16 | 1365.68 | 0.25 | -0.28 | 0.07 |
| prompt_number | 0 | 0.02 | 0.09 | 1349.54 | 0.93 | -0.04 | 0.04 |
